# Development of a remote-control system for catheterization capable of high-speed force feedback

**DOI:** 10.1007/s11548-022-02815-9

**Published:** 2023-01-23

**Authors:** Rei Takagi, Keita Osada, Akihiko Hanafusa, Motoki Takagi, Shahrol Bin Mohamaddan, Kazuyuki Mitsui, Hidenobu Anzai

**Affiliations:** 1 Department of Bio-Science and Engineering, Shibaura Institute of Technology, 307 Fukasaku, Minuma-Ku, Saitama, 337-8570 Japan; 2 Department of Advanced Machinery Engineering, Tokyo Denki University, Tokyo, Japan; 3 Fujikura Kasei Co., Tokyo, Japan

**Keywords:** Interventional radiology (IVR), Teleoperation system, Master–slave system, Electro-attractive material (EAM), Force feedback

## Abstract

**Purpose:**

There is a growing interest in minimally invasive surgery as interventional radiology (IVR), which decreases the burden on a patient. However, occupational exposure is a problem because the treatment is performed using X-ray fluoroscopic images. This problem can be solved by the development of a teleoperation system, but rapid force presentation is important to perform safe surgery. The purpose of this study is to develop a new teleoperation system that can be controlled at a high speed and can provide feedback force sensation within 20 ms delay.

**Methods:**

A master–slave-type remote-control system for catheterization was developed. A compact and high-speed force feedback system is realized using a novel electro-attractive material (EAM) device by which the resistance force is generated by the magnitude of the voltage applied. The linear and rotational movement of master is transferred to the slave device by UDP communication with the LAN cable, and the same movement is performed by two motors. The collision force of catheter or guidewire, detected by the sensor inside the slave device, is also transmitted to the master device. Two voltage-based methods for EAM: the ON/OFF and linear control methods, were implemented.

**Results:**

After the collision force is detected by the slave sensor, the voltage is applied to the EAM in the master device for an average of 10.33 ms and 15.64 ms by the ON/OFF and linear control methods, respectively. These delays are less than required 20 ms. The movement of the master was stopped by the resistance force of EAM, and that of the slave was also stopped accordingly.

**Conclusion:**

A master–slave-type remote-control system for catheterization that is capable of high-speed force feedback was developed. With a low delay, the developed system achieved the requirements of 20 ms that was aimed for this study. Therefore, this system may facilitate the realization of IVR surgery that is safe for both doctors and patients.

## Introduction

In recent years, there has been increasing interest in minimally invasive surgery, which decrease the burden on a patient. Minimally invasive surgery is a treatment method with many advantages for patients, such as faster postoperative recovery, shorter hospitalization, less postoperative pain, and less surgical bleeding. Although these advantages help to improve a patient’s quality of life, minimally invasive surgery imposes a large burden on the physician due to its difficulty. Interventional radiology (IVR) is a minimally invasive procedure in which a catheter or guidewire is inserted into the blood vessel of a patient and advanced to the lesion site under X-ray fluoroscopic imaging. During IVR, angiography is performed using a digital subtraction angiography system, which takes X-rays at 15 to 30 fps to obtain smooth images, but also increases the X-ray dose. The International Commission on Radiological Protection lists three methods of radiation protection: time, distance, and shielding [1]. As catheterization is an advanced technique, it is difficult to reduce the time required for this procedure. In addition, the surgeon wears a lead apron to prevent radiation exposure, but chronic back and neck pain can result from wearing such aprons for a long time [2]. Therefore, the development of a remote-control system is desired to prevent occupational exposure and discomfort of doctors.

In IVR, physicians rely on X-ray fluoroscopic images and tactile sensations transmitted from the catheter during surgery. Therefore, it is important to reproduce the tactile sensation of the catheter during the procedure. The maximum contact force in the linear direction transmitted to the physician is 2 N and that in the rotational direction is 50 Nm [3].

Hansen Medical SenseiX is a teleoperation system for catheters currently on the market and is used for cardiac ablation. The doctor operates the system using a joystick with three degrees of freedom and control buttons in a workspace apart from the patient. The catheter inside the manipulator also has three degrees of freedom, and the contact of the tip of the catheter can be transmitted to the doctor as vibration. In the workspace, surgical information, such as 3D mapping information and X-ray fluoroscopic images, is displayed. However, this system has disadvantages such as the difference in sensation from a normal surgical procedure due to the use of a joystick and the high cost [4, 5].

In the system developed by Omisore [6], the phantom Omni is used as the master device operated by the physician. The master device, developed by Woo [7], has five degrees of freedom. However, similar to SenseiX, these systems require physicians to deal with the different operability between the usual left-handed linear motion and right-handed rotational motion.

Guo [8] developed a master–slave system using an electromagnetic coil and a bobbin as a force presentation method. The maximum force of 0.25 N displayed by the system is almost the same as the collision force during surgery. However, the force is not sufficiently high to stop the operation. Zhang [9] developed a master–slave system using a magneto rheological fluid whose resistance force changed depending on the applied magnetic field. The disadvantages of using fluid as a force presentation device are liquid leakage, maintenance, and increased equipment size. The CathBot developed by Dadnino [10] used a motor for the force sense presentation device. In the case of using a motor, there are limitations due to backlash and the risk of runaway by malfunction.

We believe that it is important to consider operation delay in the operability of teleoperation systems. Operation delay may cause damage to the blood vessels when contact with the catheter cannot be detected. In addition, humans can sense a delay of approximately 10–20 ms [11]. Therefore, delays of more than 20 ms give the operator a sense of discomfort and cause them not to perform normally. In the system developed by Li [12], using Raspberry Pi4 as a control device, a delay of approximately 100 ms was necessary for TCP communication. In this study, a new teleoperation system was developed that can be controlled at a high speed and can provide feedback force sensation to doctors.

## System configuration

**Fig. 1:**
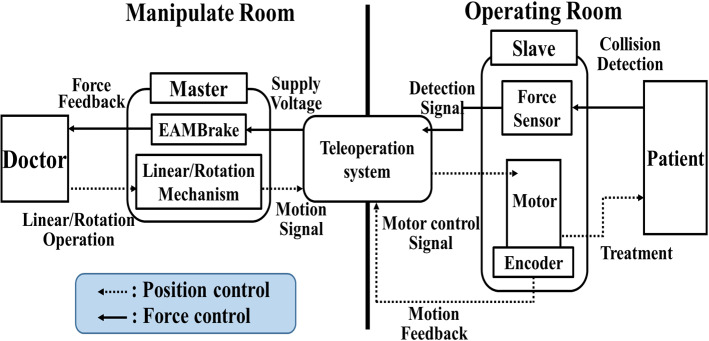
Configuration of teleoperation system

A master–slave system consisting of a master device operated by a physician and slave devices that operate catheters and guidewires (Fig. 1) was developed. The system can control the position to manipulate the catheter and force to detect collisions with the vessel wall that present force sensations to the physician. In position control, the physician performs linear and rotational movements with the master device and transmits these movements to the slave devices to operate the catheters and guidewires. In force control, the collision force between the catheter or guidewire and the vessel wall is detected by the slave device and transmitted to the master device. Thus, contact is transmitted to the physician by presenting a force sensation.

### Master device

**Fig. 2:**
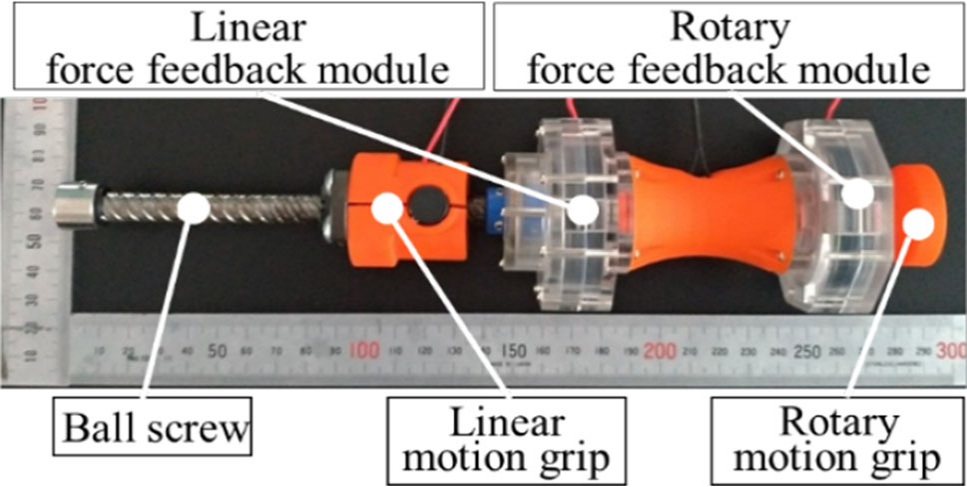
Master device

The master device is illustrated in Fig. 2. In a normal procedure, the left hand is used for linear motion, and the right hand is used for rotation. Therefore, the master device is designed such that it can be operated using the same technique that is employed in normal surgical operation. The device is compact, with a size approximately equal to a plastic bottle to facilitate the doctor operate it with a comfortable posture.

**Fig. 3:**
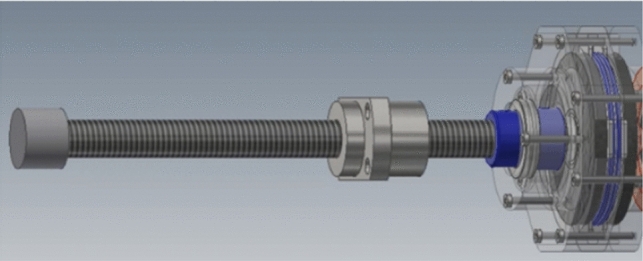
Mechanism of the master device

The position control mechanism has two degrees of freedom: linear and rotational motion. A ball screw is used as the linear motion control mechanism. Therefore, the operation in the linear direction is converted into rotational movement (Fig. 3). Both linear and rotational motions are measured by a Microtech Laboratory rotational encoder (MES-9–360-p).

**Fig. 4:**
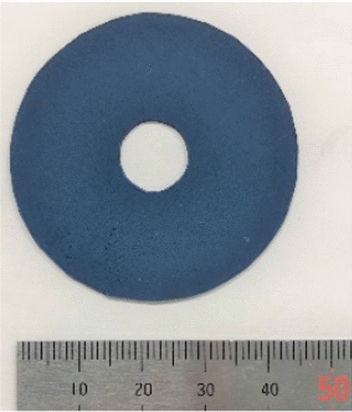
EAM sheet

**Fig. 5:**
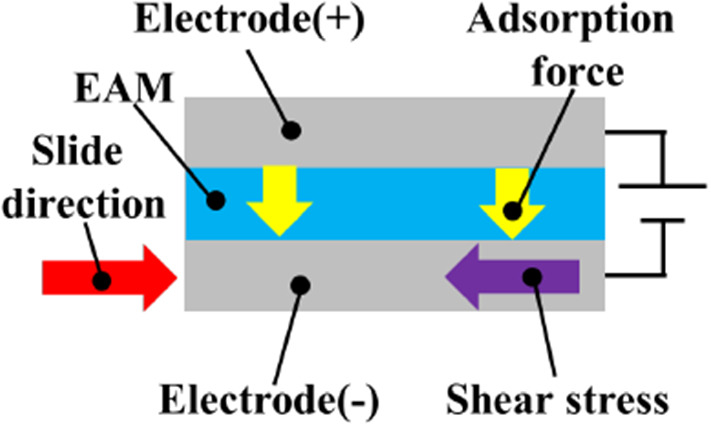
EAM configuration

A functional material called an electro-attractive material (EAM) is used to present the sense of force in the master device (Figs. 4 and 5) [13]. An EAM is a functional material in which electrorheological particles are dispersed in a sheet of silicon resin. Normally, an EAM is a smooth material, but when a high voltage is applied to an EAM sandwiched between electrodes, an adsorption reaction occurs. When a force is applied in the sliding direction while this adsorption force is generated, a shear resistance force is generated in the direction opposite to the sliding direction. This shear resistance force provides a force sensation to the doctor. The force is determined by the magnitude of the voltage applied to the EAM and the contact area with the electrode.

**Fig. 6:**
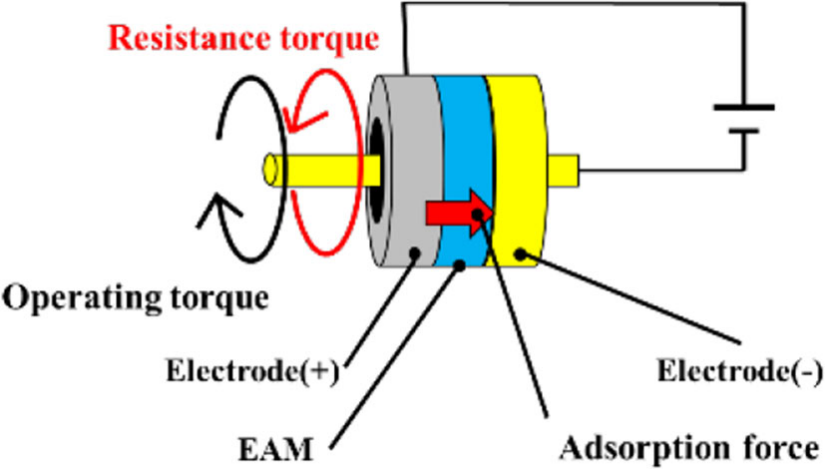
Internal structure of force feedback unit

The internal structure of the force-sensing part of the master device is shown in Fig. 6. By fixing the electrode and EAM, its contact each other in the rotational direction, the structure can effectively present the force sensation. In addition to the rotational-force-sensing part, the linear-force-sensing part is designed to contact the EAM in the rotational direction by the movement of the ball screw.

### Slave device

**Fig. 7:**
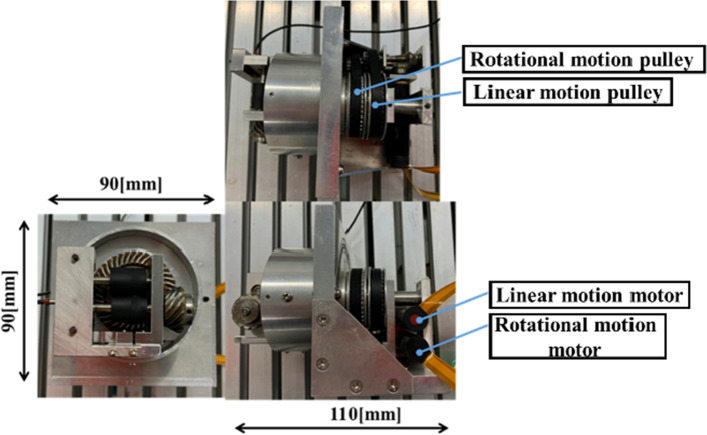
Appearance of the slave device

**Fig. 8:**
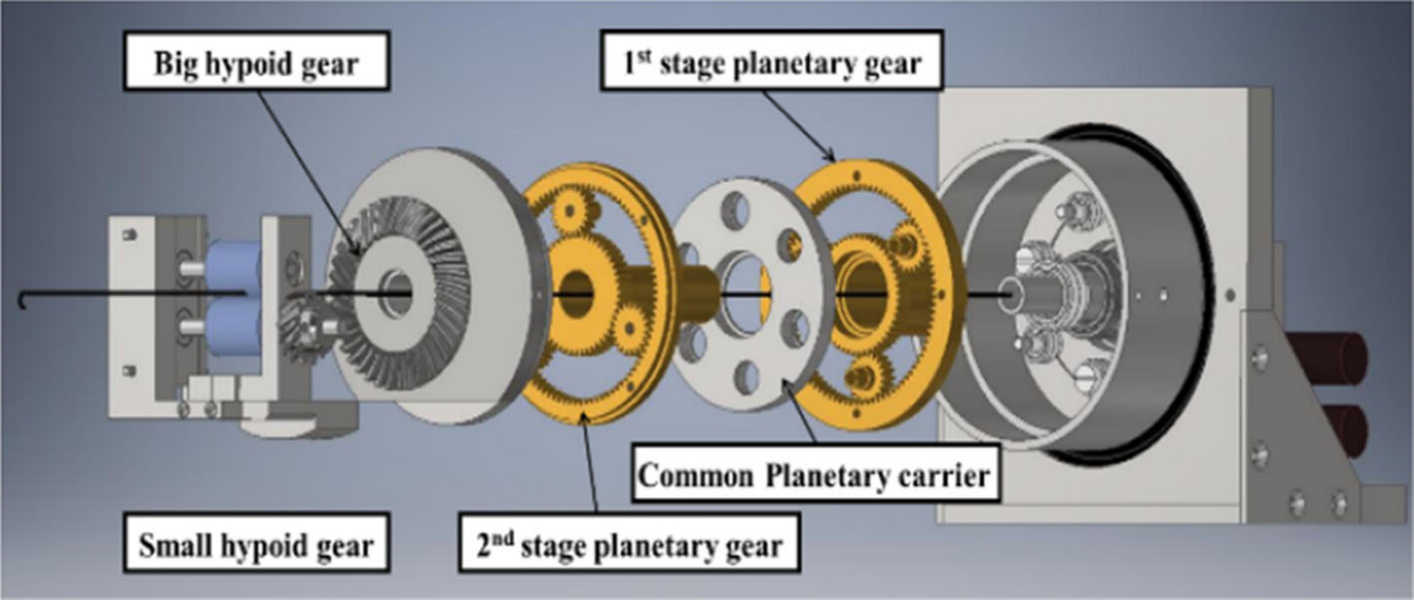
Mechanism of the slave device

The slave device is shown in Fig. 7. This device has two degrees of freedom, linear and rotational movements, and the two types of motions do not interfere. A DC motor and an encoder with a gear head manufactured by Maxon motor are used for these motions. In linear motion, the power of the motor is transmitted to the roller at the end through the miter gear, pulley, first planetary gear, common planetary carrier, second planetary gear, and hypoid gear, as shown in Fig. 8. The catheter and guidewire are sandwiched between two rollers and pulled out and in.

**Fig. 9:**
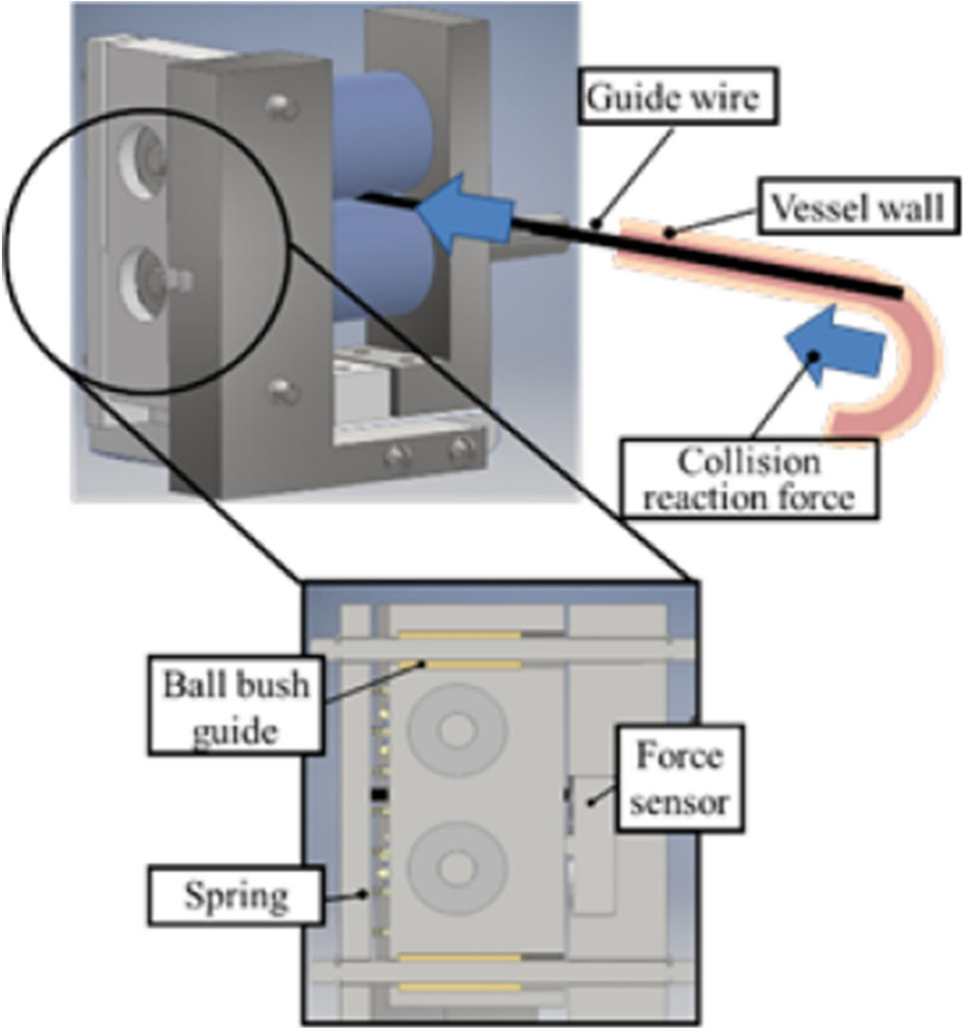
Force detection by slave device

The force detection mechanism of the slave device is shown in Fig. 9. A load of approximately 5 N is always applied to the force sensor in the linear motion direction by using a spring. When the positive direction of the collision force is applied, the initially applied force is reduced. A HSFPAR303A from Alps Alpine was used as a force sensor, and the maximum error of the detected collision force was 0.0626 N [14]. On the other hand, when a load cell (LMA-A-5 N) manufactured by Kyowa Electronic Instrument was used, the maximum error was 0.203 N, and the resolution was higher than that of the previous sensor.

### Teleoperation system

A Raspberry Pi4 Model B of Raspberry Pi Foundation is used as the control system. Each system for position and force control was programmed in the C language. As position and force control are performed on the same Raspberry Pi, threads are used for each control program to reduce the possibility of the process becoming slow. The user datagram protocol (UDP) is employed for communication with the LAN cable.

**Fig. 10:**
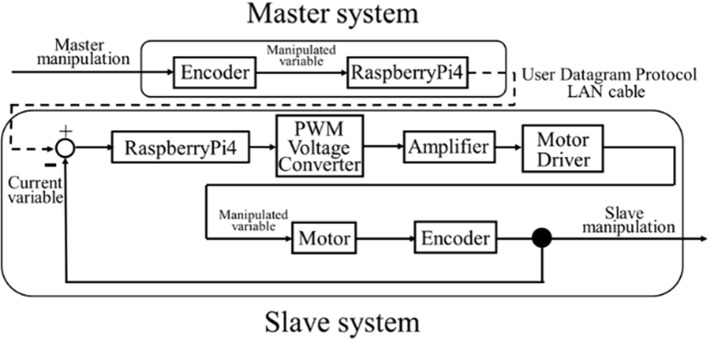
Configuration of the position control system

The position control system is shown in Fig. 10. The deviation is calculated by subtracting the value measured by the slave encoder from the target value acquired by the master encoder. Operating commands are calculated by means of the PID control method based on the deviation and transferred to the slave device.

The requirements for position control are listed below.

- Linear motion operation
  Positional accuracy: within 1 mm
  Operating speed: 70 mm/s.
- Rotational motion operation
  Positional accuracy: within 1°
  Operating speed: 1°/s
- Control delay: 20 ms or less

The results of the delay evaluation experiment of the linear position control are presented in Table 1 [15]. The average delay of the communication from the master system to the slave system is 4.63 ms for 10 trials, and the delay of the processing time of the slave system is 0.42 ms. The delay of the position control system from the start of the movement of the master device to the start of the movement of the slave device is 5.04 ms on average and 19.7 ms at the maximum, which meets the requirements. The accuracy and speed results are listed in Table 2. The steady-state deviation is 0.03 mm on average and 0.21 mm at maximum, which means that the position control system satisfies the requirements. In the sine wave input experiment, the response does not drop until an input signal of 0.25 Hz with an amplitude of 83.3 mm, and the system can provide an operation speed of up to 83.3 mm/s.

**Table 1:** Delay evaluation of linear position control system

	Communication delays [ms]	Processing delay [ms]	Position control system total delay [ms]
Average	4.63	0.42	5.04
SD	7.38	0.18	7.27
Max	19.30	0.65	19.70

**Table 2:** Accuracy and speed evaluation of linear position control system

	Steady deviation [mm]	Average speed [mm/s]
Average	0.03	104.92
SD	0.06	27.01
Max	0.21	151.04

The results of the rotational position control delay evaluation experiment are shown in Table 3 [15]. The maximum total delay is 0.70 ms. In the sine wave input experiment, the response does not drop until an input signal of 0.50 Hz with an amplitude of 180°, which means that the system can track up to 360°/s.

**Table 3:** Delay evaluation of rotational position control system

	Communication delays [ms]	Processing delay [ms]	Position control system total delay [ms]
Average	0.09	0.33	0.43
SD	0.05	0.23	0.26
Max	0.15	0.59	0.70

These results show that the position control system satisfies the requirements for both linear and rotational motions and can be controlled with high speed and accuracy (Table 4).

**Table 4:** Accuracy and speed evaluation of rotational position control system

	Steady deviation [deg]	Average speed [deg/s]
Average	0.003	430.8
SD	0.007	83.0
Max	0.013	524.4

**Fig. 11:**
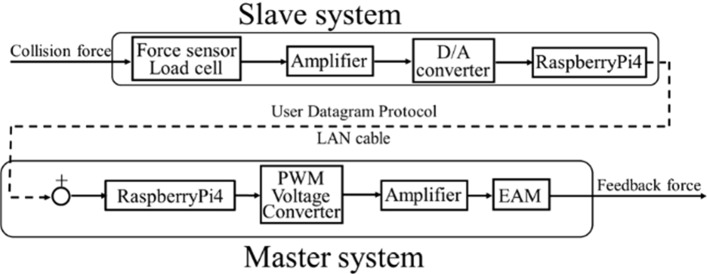
Configuration of the force control system

The configuration of the force control system is shown in Fig. 11. The contact force between the blood vessel and the catheter or guidewire, detected by the sensor inside the slave device, is transmitted to the master device via the remote-control system to present the force sensation. The delay from detection in the slave device to the presentation of the master force sensation must be maintained within 20 ms. At present, the slave device can detect the collision force in the direction of linear motion. We evaluated whether the newly developed force control system for the linear motion direction can present a force sensation within the required time. The requirements of the force control system are as follows.

- Master device presentation force
  Presentation force target value: 2 N or more
  Presentation force resolution: 0.5 N
- Slave device side detection force
  Force detection target value: 0–0.2 N
  Force detection resolution: 0.01 N
- Control delay; within 20 ms

## Evaluation of the force control system delay

The force control system transmits the collision force detected by the slave device to the master device, which then presents the force sensation to the doctor. As a delay in force presentation may impair the safety of the operation, an experiment to evaluate the system delay was conducted.

**Fig. 12:**
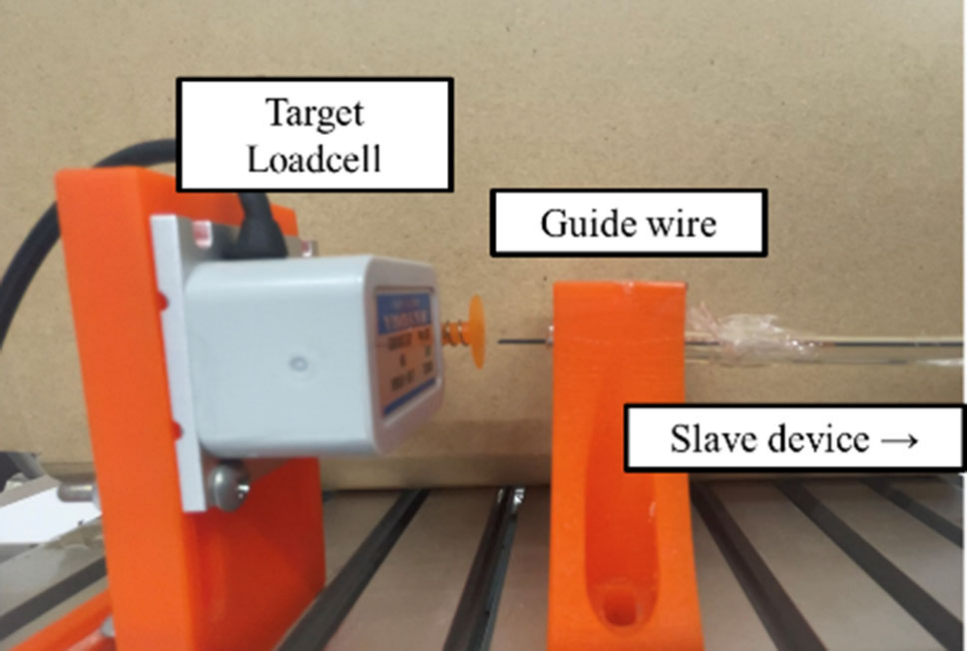
Target load cell and guide wire

**Fig. 13:**
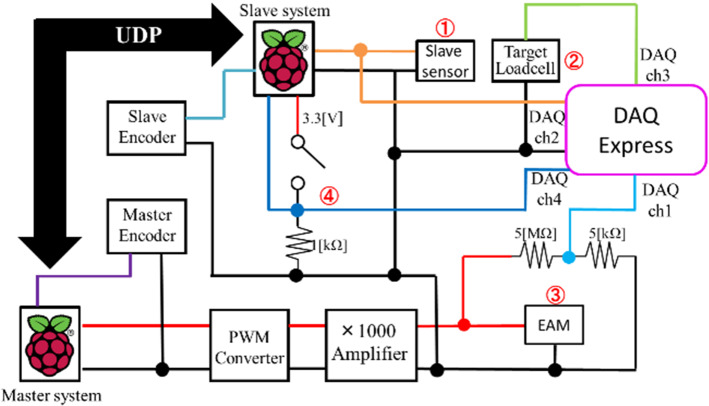
Experimental setup of electric circuits

The target load cell and the manipulated guide wire employed during the experiment are shown in Fig. 12. The subject manipulates the master device in the linear motion direction, and the slave device drives the guidewire. The configuration of the experimental setup for the electric circuits is illustrated in Fig. 13. The tip of the guidewire collides with the target load cell (② LVS-100GA Kyowa Electronic Instrument). The collision force detected by the sensor inside the slave device (①) is transmitted from the slave system to the master system via the UDP. After converting the PWM signal into an analog voltage using a PWM converter, the voltage is boosted using a 1000 × voltage booster (APM-3K10PBX by Max-electronics), and the voltage is applied to the EAM in the master device. The subject stops the operation when the force is presented by the EAM, and the master device is prohibited from operating. The voltage applied to the EAM is reduced by 1/1000 and recorded for measurement (③).

In the slave system, the value of the operation transmitted from the master device and the actual value of the operation of the slave device are recorded. In addition, the voltage values at positions ①, ②, ③, and ④ are recorded using a data acquisition system (DAQ Express by National Instrument). The slave system and data acquisition system are synchronized by the output signal (④) of the Raspberry Pi. Two control methods, ON/OFF and linear control, were tested five times each in the experiment.

### ON/OFF control method

To evaluate the control delay of the force control system, ON/OFF force presentation by the force sensor was applied. When the internal sensor of the slave device exceeded 0.51 N, 500 V was applied to the EAM of the master device. The delay from the time the target load cell exceeded the threshold until the voltage was applied to the EAM, the delay until the master device operation was stopped, and the delay until the slave device was stopped were measured.

### Linear control method

**Fig. 14:**
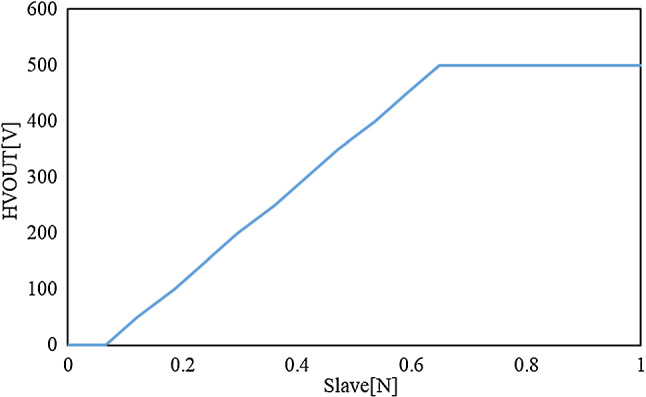
Relation between slave force and voltage applied by the linear control method

The voltage was applied to the EAM in the range of 0.065–0.647 N by the internal sensor of the slave device according to the formula shown in Eq. (1) [1], but the output voltage was limited to 0–500 V. The relationship between the slave force and applied voltage is shown in Fig. 14. The threshold slave force was set to 0.065 N, which was the starting point of the voltage applied to the EAM. The delay from the target load cell value exceeding the threshold to the voltage applied to the EAM was evaluated.

1 $$\mathrm{HVOUT} V= 859.1\cdot (\mathrm{Slave} N-0.065)$$

## Evaluation results

### ON/OFF control method

**Fig. 15:**
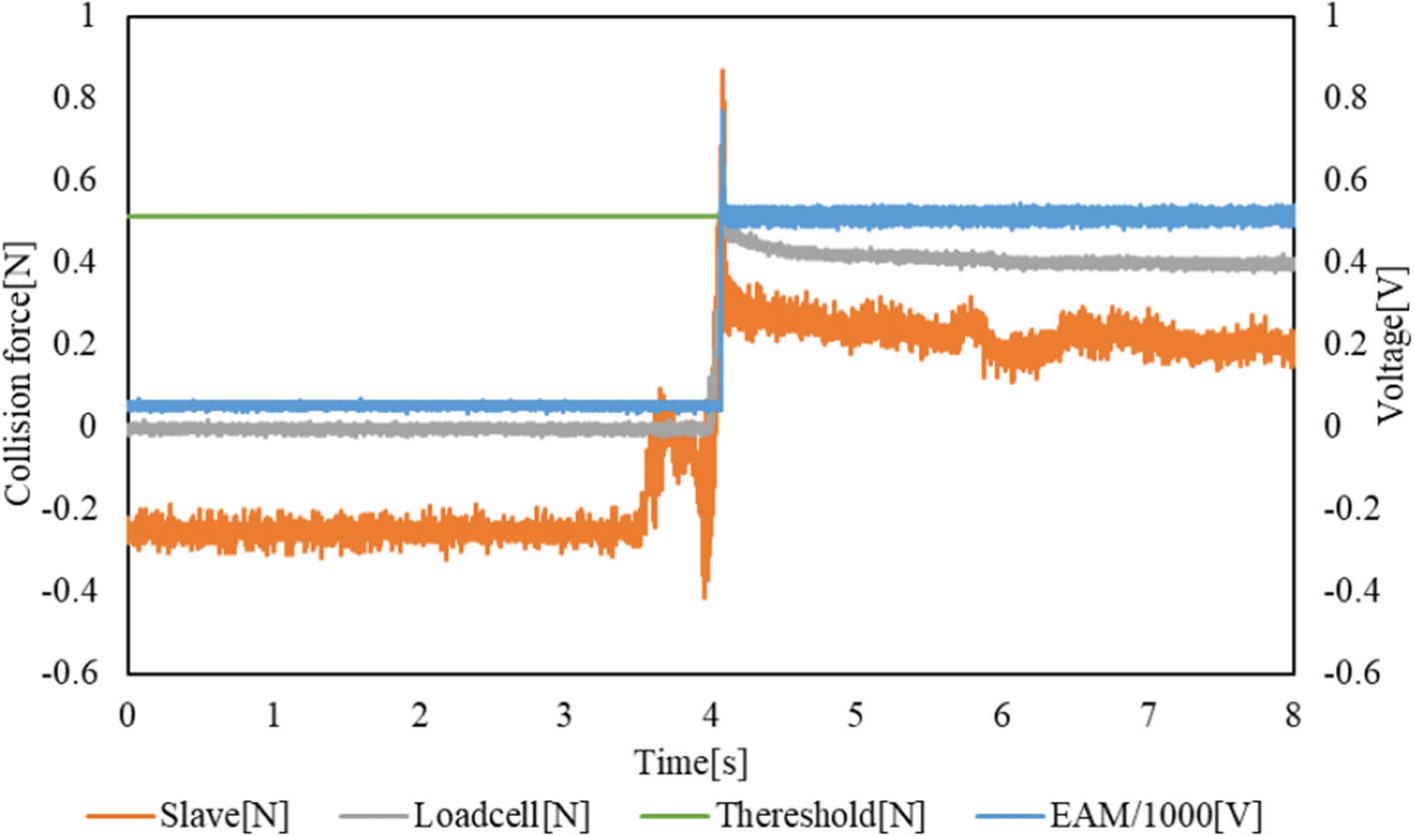
Transitions of the EAM applied voltage, impact force measured by the slave sensor, and impact force measured by the load cell with the ON/OFF control method

**Fig. 16:**
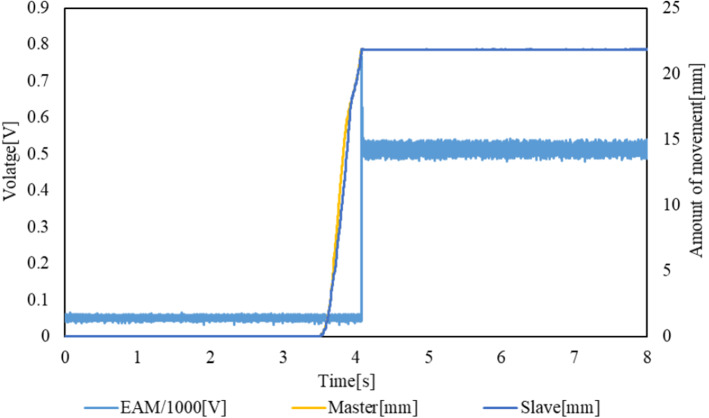
Transition of EAM applied voltage and positions of slave and master with the ON/OFF control method

The experimental results are presented in Figs. 15 and 16. The transition of the EAM applied voltage and impact force of the slave sensor and load cell are shown in Fig. 15. The voltage applied to the EAM, force obtained by the internal sensor of the slave device, and force measured by the target load cell increase simultaneously. The voltage applied to the EAM and positions of the slave and master devices are depicted in Fig. 16. The slave device was moved following the operation of the master device, and the operation of the master device was terminated after the sensed force was output, and a voltage of 500 V was applied to the EAM.

The delay between when the target load cell force exceeds the threshold of 0.51 N, and the voltage applied to the EAM is defined as the delay of the force control system (applied voltage). The delay until the operation of the master device is stopped by the presented force is defined as the delay of the force control system (movement). The delay until the slave device stops its operation is defined as the delay of the force control system (overall). Table 5 shows the maximum collision force detected by the internal sensor of the slave device and load cell in each trial. Table 6 presents the results for each type of delay explained above in each trial.

**Table 5:** Maximum collision force detected by the external load cell and internal sensor of the slave device in each trial with the ON/OFF control method

	Loadcell-max [N]	Slave-max [N]
1	0.66	0.87
2	1.19	1.77
3	0.72	0.76
4	0.84	0.75
5	1.18	1.54
Average	0.92	1.14
SD	0.25	0.48

**Table 6:** Delay of each type with the ON/OFF control method

	Force control system delay (Voltage) [ms]	Force control system delay (Movement) [ms]	Force control delay (Total) [ms]
1	3.83	2.19	6.97
2	6.83	7.03	379.65
3	17.00	15.73	18.44
4	5.33	6.96	13.40
5	18.66	28.99	103.85
Average	10.33	12.18	104.46
SD	6.95	10.59	158.84

The results of the 5 trials performed confirm that the average delay until the voltage is applied to the EAM is 10.33 ms. Even the maximum value of 18.66 ms is less than 20 ms, which means that the delay meets the requirements. In addition, the average delay until the operation of the master device is stopped is 12.18 ms, which again is less than 20 ms. The total average delay is large, at 104.46 ms. In particular, the delays are larger in the second and fifth trials. One reason for this result is that the position is controlled by PID control in this system. However, if the operation speed of the master device increases, the slave device cannot follow it and deviation remains. Therefore, even if the operator stops operation of the master device after the force is applied, the slave device continues to move until the deviation disappears. However, if the movement stops when the collision is detected even if the deviation remains, the catheter or guide wire can be stopped safely.

Another reason is that there will be differences between the forces measured by the sensor in the slave device and in the load cell, even after calibration. If there is an error in the measured force, the threshold measurement may be delayed. On average, the collision force detected by the internal sensor of the slave device is larger than that detected by the target load cell, as shown in Table 5, and the slave device can be stopped more quickly and safely.

### Linear control method

**Fig. 17:**
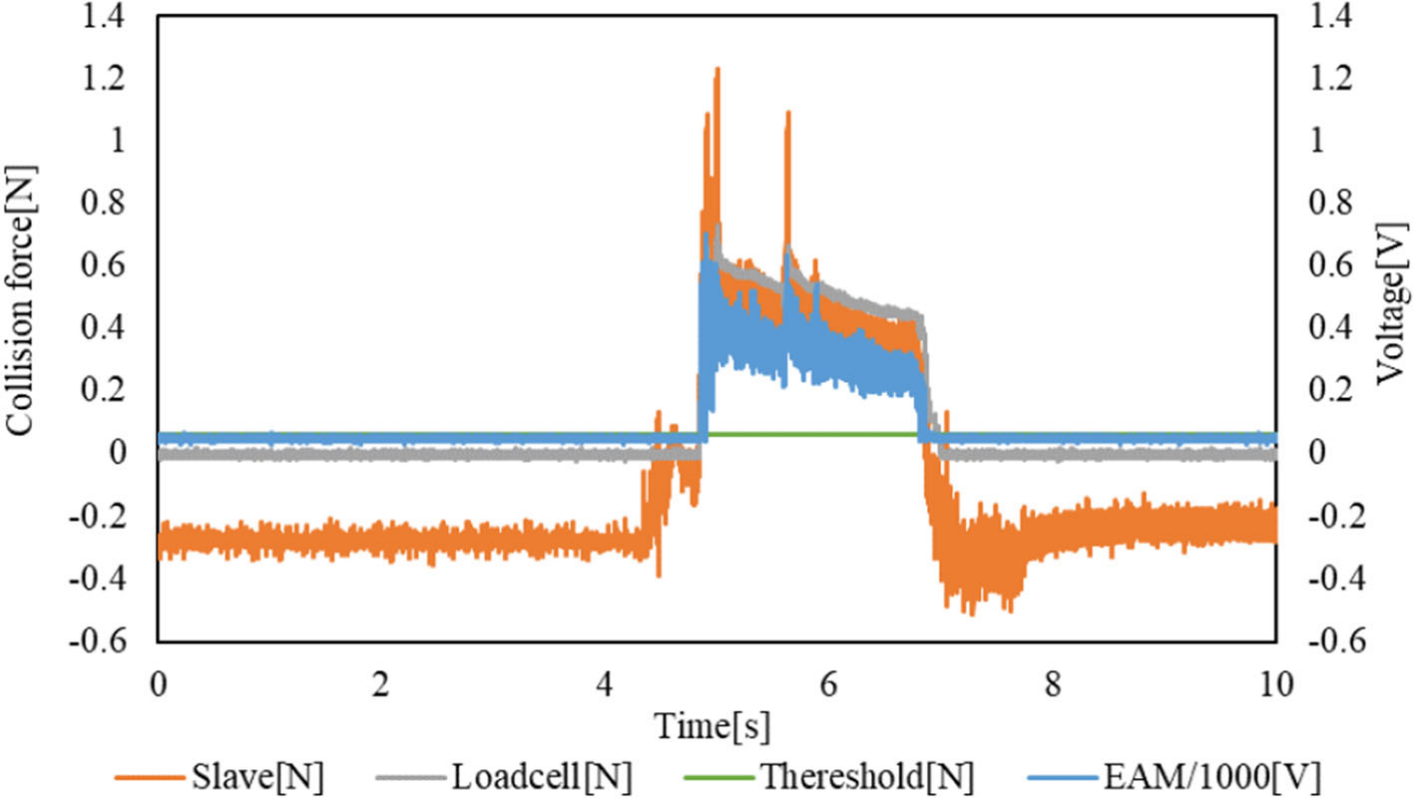
Transitions of EAM applied voltage, impact force on the slave sensor, and impact force on the load cell with the linear control method

**Fig. 18:**
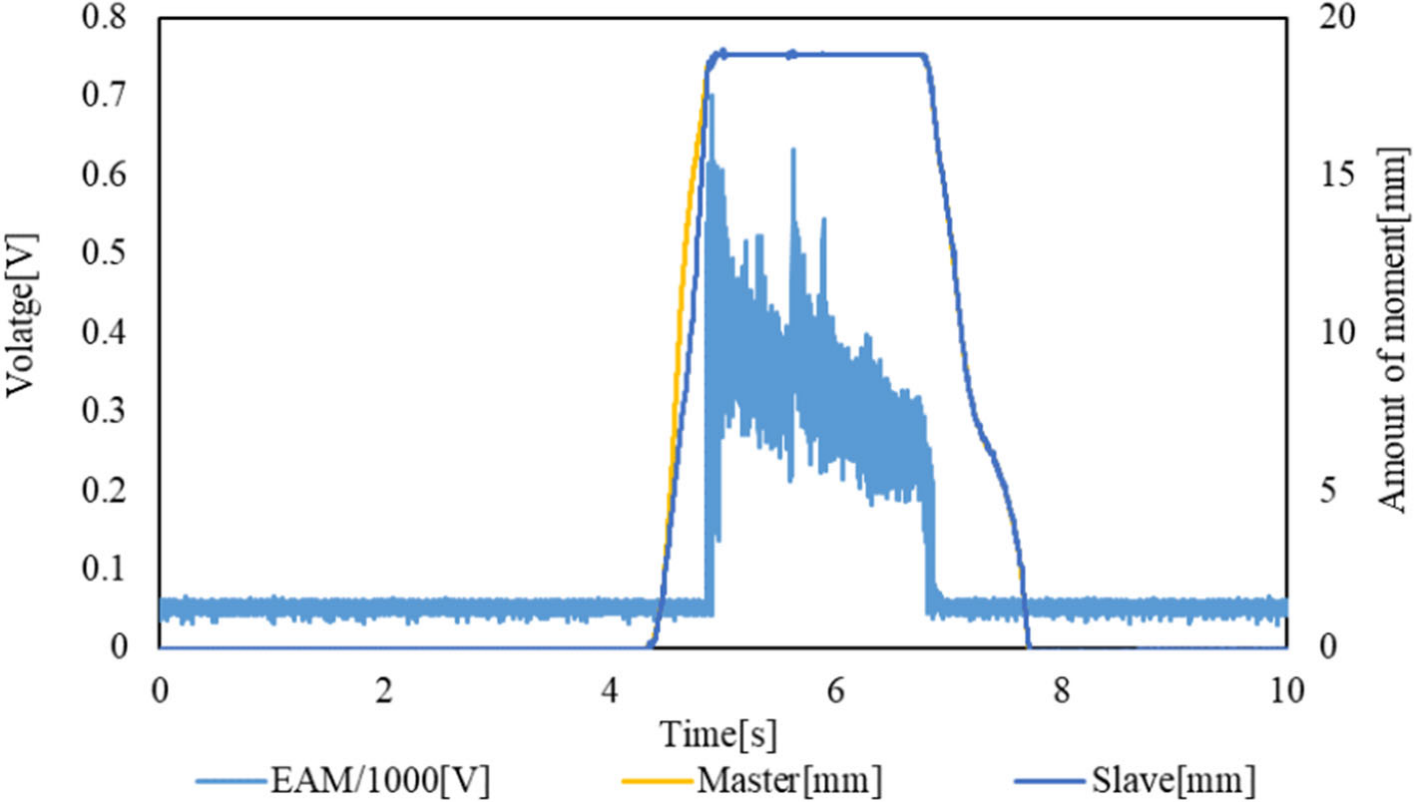
Transitions of EAM applied voltage and positions of slave and master with the linear control method

The experimental results are presented in Figs. 17 and 18. The transition of the EAM applied voltage and the impact forces of the slave sensor and load cell are shown in Fig. 17. The voltage applied to the EAM, force obtained by the internal sensor of the slave device, and force on the target load cell increase simultaneously, as in the case of the ON/OFF control method. It can also be confirmed that the slave device is operated following the operation of the master device. The value measured by the internal sensor of the slave device increases after the guidewire collides with the load cell. The transitions of the EAM applied voltage and the positions of the slave and master are shown in Fig. 18. The voltage applied to the EAM increases, and the movement of the master device stops. However, the value measured by the sensor of the slave device decreases, and the voltage applied to the EAM also decreases. Thus, the master device can be operated again, and the slave device can pull back the guide wire to the original position.

The delay from the time at which the collision force detected by the load cell exceeds the threshold value of 0.065 N until the voltage is applied to the EAM is defined as the (voltage) delay of the force control system. The delay and maximum collision force detected by the external load cell and internal sensor of the slave device in each trial are listed in Table 7. The rise times of the signals generated by the load cell and sensor built into the slave and the master operation speed in each trial are shown in Fig. 18. The average delay in the five trials conducted is 15.64 ms, which satisfies the requirement of 20 ms. However, in the fourth trial, the delay exceeds the requirement.

**Table 7:** Delay until the voltage is applied to EAM and maximum collision force detected by the external load cell and internal sensor of the slave device in each trial with the linear control method

	Force control system delay (Voltage) [ms]	Loadcell-max [ms]	Slave-max [ms]
1	11.27	0.55	0.77
2	13.80	1.23	1.49
3	12.60	1.20	1.56
4	23.03	0.77	0.79
5	17.50	1.10	1.57
Average	15.64	0.97	1.24
SD	4.74	0.29	0.42

We attempted to evaluate the relationship between the applied voltage and generated feedback force. Using the table driven by the motor, the master device was pulled by the wire, and the tension force of the wire that represented the feedback force was measured by a tension sensor. Until 300 V is applied, almost no force is detected. However, when 500 V is applied, the feedback force is approximately 5 N. Therefore, when the slave device collides with the target, more than 500 V is applied to the EAM, and more than 5 N feedback force is generated. The feedback force is sufficient to stop the movement of the master device.

When the guidewire contacts the target load cell, the values of the target load cell and slave sensor increase rapidly, as shown in Fig. 17 and Table 8. It takes only 43.52 ms from the time at which the internal sensor of the slave device starts to detect a collision until it reaches the maximum value. In this experiment, the master device is operated at a slow speed of 44.86 mm/s on average. Therefore, the voltage applied to the EAM increases to the maximum value, as shown in these experimental results, which is practically the same as that obtained with the ON/OFF control method. In this experiment, we attached a spring to the target load cell to make it similar to an elastic body. However, it seems that the spring constant is too large. It is necessary to check the rise of the internal sensor of the slave device using an actual blood vessel in the future to evaluate which control method is better.

**Table 8:** Rise times of the signals obtained by the load cell and sensor built into the slave and master operation speed in each trial with the linear control method

	Loadcell-rise time [ms]	Slave-rise time [ms]	Operation speed [mm/s]
1	34.07	27.20	31.99
2	64.67	51.23	38.57
3	75.44	61.17	49.22
4	43.07	29.00	47.75
5	55.84	49.01	56.80
Average	54.62	43.52	44.86
SD	16.52	14.82	9.69

### Comparison between ON/OFF and linear control methods

The delay until the voltage is applied to the EAM is smaller with the ON/OFF control method than with the linear control method. However, both methods satisfy the requirements specified. The force sensation is presented to the surgeon to enable not only collision detection, but also reproduction of the actual force felt during an operation, such as the friction inside a vessel. To realize such a feedback force, we believe that the linear control method is effective. To utilize the advantages of both methods investigated, stepwise control should be attempted that can provide force sensation to the doctor, as in the linear control method, and reduce the delay, as in the ON/OFF control method.

## Conclusion

In this study, a master–slave-type remote-control system for catheterization was developed. The key feature of the system is that the high-speed force feedback system in the linear motion direction is included to present a force sensation to the surgeon. After the collision force was detected by the slave sensor, the voltage was applied to the EAM in the master device for averages of 10.33 ms and 15.64 ms when the ON/OFF and linear control methods were used, respectively. These delays are less than 20 ms and therefore meet the requirements specified. However, it is necessary to stop the movement of the slave device after the collision is detected to construct a system with higher safety.

In the future, it will be necessary to examine a stepwise control method to improve the operability of surgeons while maintaining a short reaction delay. The system should be evaluated by doctors, and it should be checked which control method is suitable. It is also necessary to design a mechanism to detect the contact force in the rotational direction, which has not yet been developed. In addition, we would like to evaluate whether it is possible to detect the collision force of the guidewire in a complicated path using a modeled or an actual blood vessel that is close to that in an actual surgical environment. The system may facilitate realization of safe IVR surgery for both doctors and patients.
